# Controlling the Interaction between Starchy Polyelectrolyte Layers for Adjusting Protein Release from Nanocapsules in a Simulated Gastrointestinal Tract

**DOI:** 10.3390/foods11172681

**Published:** 2022-09-02

**Authors:** Yingying Li, Ying He, Xiaoxi Li

**Affiliations:** Ministry of Education Engineering Research Center of Starch and Protein Processing, Guangdong Province Key Laboratory for Green Processing of Natural Products and Product Safety, School of Food Science and Engineering, South China University of Technology, Guangzhou 510640, China

**Keywords:** deposited layer number, release kinetics, in vitro release property

## Abstract

Orally delivered bioactive proteins face great challenges in the harsh environment of the upper gastrointestinal tract (GIT) in the field of functional foods based on bioactive proteins. Therefore, it is necessary to design carriers and delivery systems that have the potential to overcome the problem of lower bioaccessibility for protein cargoes. In this work, we present a starchy oral colon-targeting delivery system, capable of improving the release profile of the protein cargoes. The starchy oral colon-targeting delivery system was fabricated using layer-by-layer assembly of starchy polyelectrolytes (carboxymethyl anionic starch and spermine cationic starch) onto the surface of protein nanoparticles via electrostatic interaction. The dynamic change in the interaction between the starchy polyelectrolytes affected the shell aggregation structure and determined the release kinetics of nanocapsules in the GIT. Specifically, the stronger interactions between the starchy layers and the thicker and more compact shell layer kept the nanocapsule intact in the simulated gastric and intestinal fluids, better-protecting the protein from degradation by digestive fluids, thus avoiding the burst release effect in the SGF and SIF. However, the nanocapsule could quickly swell with the decreasing molecular interactions between starchy polyelectrolytes, increasing protein release (63.61%) in the simulated colonic fluid. Therefore, release behaviors of protein cargoes could be appropriately controlled by adjusting the number of deposited layers of pH-sensitive starchy polyelectrolytes on the nanocapsule. This could improve the bioaccessibility of oral targeted delivery of bioactive proteins to the colon.

## 1. Introduction

Bioactive proteins are attracting growing interest for their potential health benefits. This has led to a recognized consensus that food fortified with bioactive proteins will be beneficial in preventing chronic diseases, such as type 2 diabetes, obesity, hypertension, and cardiovascular disease [1,2]. Therefore, considerable efforts have been made in the encapsulation and oral delivery of bioactive proteins, especially in food-grade delivery systems [3,4,5,6]. However, the bioaccessibility of protein is greatly challenged by harsh barriers in the upper gastrointestinal tract (GIT) (i.e., extreme gastric pH, intestinal protease, ionic strength, and long transit time). Colloidal delivery systems show great potential to encapsulate and protect protein cargoes during oral transmission [7,8]. These applications include hormones, enzymes, and probiotics [9]. Various biopolymers, such as alginate [5], pectin [10], modified starch [6], and cellulose [3,11] have been developed for specific strategies. Although the potential for colloidal colonic delivery has been demonstrated in numerous studies on improving bioavailability and targeted delivery, the efficiency of current food-grade colloidal delivery still does not meet expectations. Additional work is necessary to develop a more scientific delivery system and receive a better release profile for these bioactive proteins.

During transit in the GIT, the colloidal shell materials are challenged by variation in pH (1.0–3.0 in the stomach and near neutral in the small intestine and colon) and various digestive enzymes (pepsin in the stomach and pancreatin in the small intestine) in specific locations, which results in structural changes and makes them shrink, swell, or erode, affecting the release of the protein cargo from the core. This is challenging but also offers the potential for structuring a colloidal system. Theoretically, to control the release rate of the protein cargo from the colloidal capsule, the key is to scientifically construct the outer shell material based on the GIT features. The architecture and properties of the systems can be scientifically controlled using layer-by-layer self-assembly [12,13,14,15]. The release behaviors in the GIT can be controlled by the deposition of pH-sensitive polyelectrolytes on the nanocapsule. Furthermore, the release rate can be appropriately adjusted by controlled compactness of the shell, as related to control of the polyelectrolytes’ substitution degree of modified groups [6], molecular weight [6], or deposition amount [16]. Therefore, controlling the interaction of deposited polyelectrolytes is a sound strategy for regulating the release properties of nanocapsules in the GIT.

The relationships between the changes in the deposited shells’ structure and release behaviors in the GIT are highlighted in this work. As mentioned above, an appropriate shell structure should facilitate efficient oral delivery. Starch polyelectrolyte [17,18], a green, safe, cheap, and biodegradable food-grade polymer has been widely explored for encapsulating and delivering functional proteins, such as insulin, lysozyme, and bovine serum albumin [18,19,20]. In our previous study, insight into the assembly behavior of starchy polyelectrolytes on the protein surface was gained by modifying properties of the polyelectrolytes [6,16]. For instance, adjusting the substitution degree of modified groups [6], adjusting molecular weight [6], and changing the ratio between the oppositely charged polyelectrolytes [16] improved the colon-targeting property of protein cargoes in nanocapsules. Furthermore, this can improve the controlled-release properties based on the scientific adjusting of release kinetics of nanocapsules in the dynamic GIT, giving valuable information for designing a colon-targeted delivery system. However, there is a relatively limited understanding of this process. Therefore, we investigated the release kinetics of the multi-layer starchy nanocapsules in the GIT and revealed the relationship between the dynamic change of shell structure and release kinetics, with the aim of adjusting the release profile of the protein cargo.

This study will provide insight into the effect of the interaction of a starchy deposited shell on the release kinetics in the GIT environment, in turn allowing design of a more scientific colon-targeted delivery system. With insulin as a model bioactive protein, we fabricated different layered nanocapsules with oppositely charged starchy polyelectrolytes: carboxymethyl anionic starch (CS) and spermine cationic starch (SS). The in vitro release from nanocapsules was carried out in simulated digestive fluids. Additionally, the release profiles were fitted to the Korsmeyer–Peppas potential equation to investigate the drug-release rate and release kinetics in the GIT. The structural characterization of the starch polyelectrolytes in the simulated GIT was also determined by dynamic light scattering and nano-isothermal titration calorimetry (ITC). Additionally, the changes in surface potential, size distribution, and aggregation structure of quintet-layer nanocapsules in simulated GIT were investigated with dynamic light scattering and small-angle X-ray scattering (SAXS), revealing its controlled-release mechanism. These results will provide a valuable reference for designing a colon-targeted delivery system based on controlling the release kinetics via adjusting the deposited layers of starch polyelectrolytes on the nanocapsule surface.

## 2. Materials and Methods

### 2.1. Materials

Corn starch was obtained from Hebei Huachen Starch Sugar Co., Ltd. (Shijiazhuang, Hebei Province, China). The CS (DS = 0.161, M_w_ = 1.115 × 10^5^ g/mol) and SS (DS = 0.793, M_w_ = 3.850 × 10^4^ g/mol) were synthesized according to our previously published method [5,20]. Insulin was from Sigma-Aldrich Co., Ltd. (St. Louis, MO, USA). A bicinchoninic acid protein assay kit was supplied by New Probe Bioscience & Technology Co., Ltd. (Beijing, China). Pepsin (EC 3.4.23.1, from porcine) and pancreatin (EC 232-468-9, 8 × USP, from porcine pancreas) were obtained from Sigma-Aldrich Co., Ltd. All other chemicals and reagents were of analytical grade and from Sigma-Aldrich Co., Ltd.

### 2.2. Preparation of the Multi-Layer Nanocapsule-Encapsulated Protein

Nanocapsule-encapsulated proteins with different layers were prepared by the layer-by-layer assembly method (25 °C) with PBS (pH 3.0) as the assembly medium according to our reported method [6,16] with some modifications. Protein solution (2.5 mg/mL) was slowly dripped into the CS solution at a 1:4 ratio for 2 h. The mixed solution was incubated at 25 °C with mild stirring for 2 h. After centrifugation at 10,614× *g* for 10 min, the residue was washed with distilled water to get a single-layer nanocapsule-encapsulated protein (IN). The single-layer nanoparticles were redispersed in the PBS (1:1 volume ratio) and then the SS solution was added into it at a quality ratio of 1:4 (CS:SS). The complex was centrifuged for 10 min (10,614*× g*) to precipitate the double-layer nanocapsules. In the same way, triplet-, quartet-, and quintet-layer nanocapsule-encapsulated proteins were assembled through repeating these above layer-by-layer process. The encapsulation efficiency (EE) of protein in the nanocapsules was determined by our reported method [18]. The EE of all nanocapsules determined in this work was 58.68%.

### 2.3. Structural Properties of the Multi-Layer Nanocapsule

The ζ-potentials of CS and SS were measured using a Zetasizer Nano-ZS (Malvern Instruments, Malvern, UK) equipped with a 4 mW helium/neon laser at a wavelength output of 633 nm and a backscattering angle of 173° at 25 °C [21]. The samples were diluted with 0.1 M PBS, with different pH values (pH 1.2, 3.0, 4.0, 5.0, 6.0, 6.8, and 7.2) and placed in the specific shell for ζ-potential analysis. Each measurement was performed in triplicate.

### 2.4. In Vitro Release

To investigate the release behaviors of the starchy nanocapsules in simulated GIT, a dissolution-rate test apparatus (RCZ-8B, Tianfa Co., Ltd., Tianjin, China) and ultraviolet spectrophotometer (UV-2102PC, Unico Instrument Company, Shanghai, China) were employed to determine EE and accumulation rate of protein according to Situ et al. [18]. All in vitro measurements were conducted at 37 °C with gentle stirring (100 rpm). Nanocapsules were incubated in simulated gastric fluid (SGF) (0.2 g of NaCl, 7.0 mL of HCl, and 3.2 g of pepsin in 1 L with pH 1.2) for 2-h, followed by 6-h incubation in simulated intestinal fluid (SIF) (6.8 g of KH_2_PO_4_, 190 mL of NaOH, and 10.0 g of pancreatin containing 1% *w*/*w* pancreatin in 1 L with pH 6.8), and another 26-h incubation in simulated colonic fluid (SCF) (0.1 M PBS in 1 L with pH 7.2). Each 5 mL of sample was collected at intervals to investigate the accumulated release of protein in vitro.

### 2.5. Characterization of the Quintet-Layer Nanocapsule in Simulated GITs

Evaluating release behaviors of protein in the GIT, the charge, particle size, aggregation structure, and interaction of wall materials in the quintet-layer nanocapsule were investigated for different simulated GIT pH values (SGF pH 1.2, SIF pH 6.8, and SCF pH 7.2).

The ζ-potential and size distribution of the quintet-layer nanocapsule was determined with the Nano-ZS in PBS buffer solution (pH 1.2, 6.8, and 7.2) at 25 °C [21].

The aggregation structure of the quintet-layer nanocapsule was measured by small-angle X-ray scattering (SAXS) (SAXSess, Anton-Paar, Graz, Austria) with Cu Kα radiation (0.1542 nm wavelength) at 40 kV, 50 mA, and exposure time of 30 min [22]. The samples were dispersed in simulated GIT fluids and used to fill a capillary at 37 °C. Then, the IP Reader software collected the data in a Perkin Elmer Storage Phosphor System, which was analyzed using SAXSquant 2D and SAXSquant 3.0 software (Anton-Paar, Graz, Austria).

The interaction between CS and SS molecules was measured using ITC (NANO ITC, Newcastle, TA, USA) according to previous studies [6,23] with some modifications. The CS and SS were dissolved in the PBS (0.01 M, pH 3.0) and equilibrated at 25 °C. The SS solution was titrated into the CS solution (10 mg/mL) every 300 s, with PBS (0.01 M, pH 3.0) as a blank reference.

### 2.6. Statistical Analysis

Experiments were carried out in triplicate and are presented as mean ± SD (*n* = 3). Statistical analysis was performed using IBM SPSS statistics version 21.0 (IBM, Armonk, NY, USA). Analysis of variance (ANOVA) was followed by Tukey’s HSD test to compare the treatments. A value of *p* < 0.05 was set as significant.

## 3. Results

### 3.1. Characterization of Nanocapsules with Various Layers

**Figure 1:**
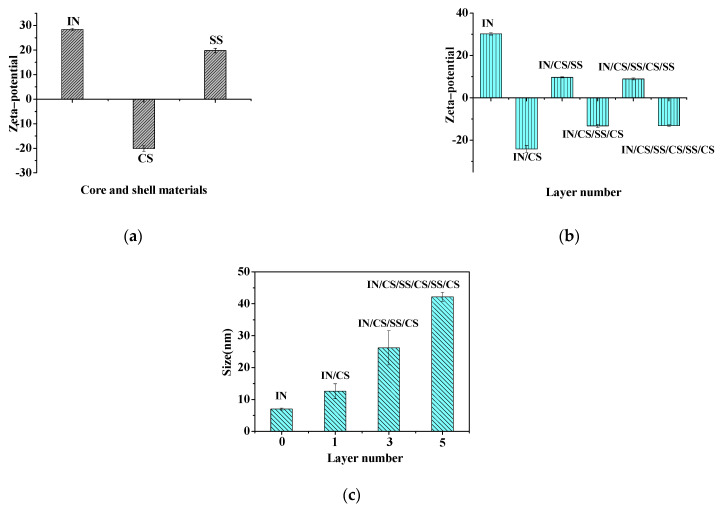
Zeta−potentials of shell materials (**a**) and multi−layer nanocapsules (**b**), and size of multi−layer nanocapsules (**c**). Abbreviations: IN, insulin; CS, carboxymethyl anionic starch; and SS, spermine cationic starch.

The surface charge is one of the most effective ways to monitor nanocapsule fabrication during the layer-by-layer assembly. In this study, the ζ-potential of the nanocapsules was measured following the deposition of each layer. The assembly pH was chosen as pH 3.0, because CS (negatively charged) and SS (positively charged) had a maximum potential difference of 40 mV at pH 3.0 (Figure 1a). This allowed them to form a complex on the surface of the protein cargoes with electrostatic interaction, improving the potential of resistance of the nanocapsule toward the enzyme during GIT transit. Furthermore, the CS and SS are both pH-sensitive polymers that offer colon-targeted potential. Specifically, the -COOH of CS cannot be ionized in the acidic stomach and presents in a gel state, inhibiting erosion by the stomach fluid [24,25]. However, there may be stronger interaction between CS and SS at the intestinal pH (6.8), and the compact deposition may reduce the permeation of the intestinal fluid [16]. Thus, improving the protein release efficiency in the lower GIT via scientifically adjusting the structure between the CS and SS shows great potential. Based on this hypothesis, we designed a nanocapsule with the CS and SS as the outer shell by varying the layer number and so changing the layer interaction. The nanocapsule charge alternated between positive and negative charges as the number of layers increased from single to quintet (Figure 1b). This indicated that nanocapsules were formed by the alternate deposition of polyelectrolytes [21] (CS (negative charge) and SS (positive charge)) on the protein surface to form the multi-layer nanocapsules. The surface charge of the triplet- and quintet-layer nanocapsules decreased compared to the single one, which was also caused by the interaction with the positively charged SS.

Hydration diameters of the insulin and nanocapsules of different layers (single-, triplet-, and quintet-layer) were also evaluated (Figure 1c). The size of the nanocapsules increased from 13 to 42 nm when the layer number increased from single to quintet (Figure 1b), indicating that more modified starch had been deposited on the protein surface by electrostatic interaction, increasing the shell thickness. Thus, the multiple-layer nanocapsules were synthesized using layer-by-layer assembly in this study.

### 3.2. In Vitro Release Profile

**Figure 2:**
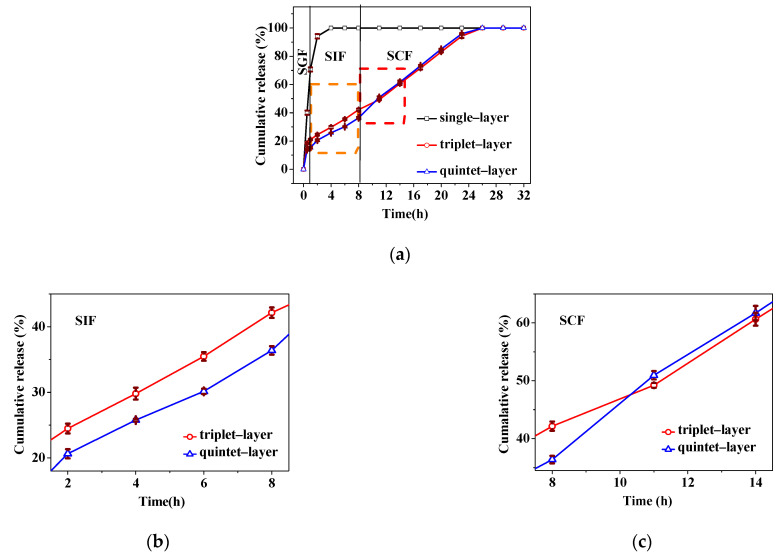
The release profile of protein from nanocapsules in the whole GIT (**a**), the SIF (**b**), and the SCF (**c**). Abbreviations: SGF, simulated gastric fluid; SIF, simulated intestinal fluid; and SCF, simulated colonic fluid.

Protein-loaded nanocapsules with different layers (single, triplet, and quintet) were treated with SGF, SIF, and SCF to determine the release behavior of the starchy nanocapsules (Figure 2a). The layer number significantly influenced protein release. For example, a single-layer nanocapsule showed the fastest release rate in the upper GIT compared to triplet- and quintet-layer nanocapsules, and around 98% of the IN was released in the stomach. In this case, the protein release rate was too fast in the SGF to reach the colon. For the triplet-layer nanocapsule, there was about 43% IN release in the upper GIT and 57% IN release in the SCF. When the layer increased to quintet, there was the smallest IN release in the upper GIT (36%) and most IN was sustainably released in the lower GIT (64%), presenting a better release property (Figure 2b,c). Therefore, the quintet-layer nanocapsule was more suitable for the release of IN, and the release behavior could be controlled via modifying the layer number.

### 3.3. Release Kinetics of Nanocapsules in Simulated GIT

The release rate of nanocapsules was determined by a zero-order model with a high R^2^ > 0.965 (Table 1). The K_0_ value is generally related to the release rate, with a higher value indicating a faster release rate [26]. The single-layer nanocapsules showed the most rapid release rate with the maximum K_0_ value of 34.267 in the SGF. As the number of layers increased to triplet, the K_0_ values decreased significantly to 4.007 in the SGF and 2.935 in the SIF, suggesting that the release rate was much slower than that of the single-layer nanocapsule in the upper GIT. With further increase to quintet-layer, there were smaller K_0_ values compared to the triplet-layer in the SGF and SIF; however, K_0_ values increased slightly and were more than those of the triplet-layer^,^ in the SCF. To sum up, the quintet-layer nanocapsule showed the best controlled-release properties for IN in the GIT.

To determine the protein dissolution mechanism, controlled drug-release data were fitted to the Korsmeyer–Peppas potential Equation (1) [27,28]:

M_t_/M_ꝏ_ = Kt ^n^ (1)

where M_t_ and M_ꝏ_ are the amounts of drug released cumulatively at time t and at the infinite time (the maximum released amount found of the release curves), respectively; K is a constant related to the drug release; and *n* is the diffusion exponent describing the drug-release mechanism. Fick diffusion is dominant when *n* ≤ 0.43, indicating that protein release is controlled by diffusion. In addition, the diffusion mechanism is non-Fickian when 0.43 < *n* ≤ 0.85, which is attributed to drug diffusion and erosion. Dissolution is mainly driven by erosion when *n* > 0.85 [26,27].

**Figure 3:**
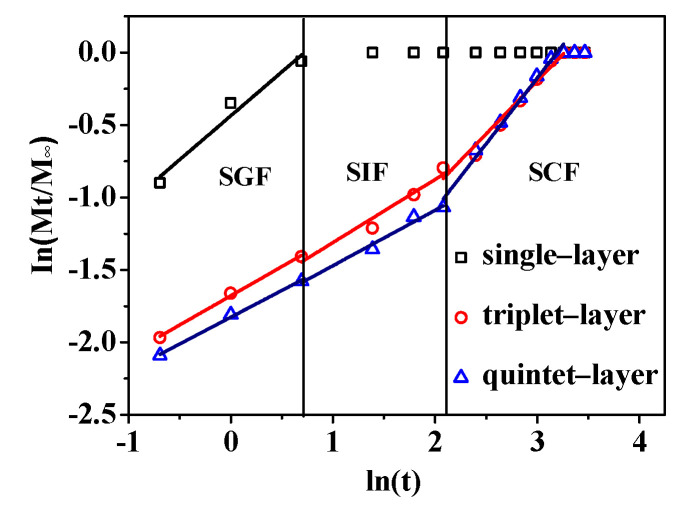
ln(Mt/M∞) vs. ln(t) curve of protein release. Abbreviations: SGF, simulated gastric fluid; SIF, simulated intestinal fluid; and SCF, simulated colonic fluid.

The release curves of nanocapsules were well fitted to the Korsmeyer–Peppas model with R^2^ > 0.962 (Figure 3 and Table 1). This clearly showed that the drug-release mechanism followed Fickian and non-Fickian diffusion mechanisms, where drug diffusion or polymer relaxation played important roles in protein release. The constant *n* differed in the nanocapsules with different layers and digestive fluids, indicating different dissolution mechanisms. The value of *n* related to the single-layer nanocapsule was 0.606 in the SGF, indicating that the drug-release mechanism was controlled by diffusion and polymer relaxation. Up to the triplet-layer nanocapsule, values of *n* indicated that the drug-release mechanism was Fickian in the SGF (*n* = 0.403) and non-Fickian in the SIF (*n* = 0.435) and SCF (*n* = 0.732). However, for the quintet-layer nanocapsule, the drug-release mechanism followed a Fickian distribution in the SGF (*n* = 0.403) and SIF (*n* = 0.403) and non-Fickian in the SCF (*n* = 0.403). 

For the single-layer nanocapsule, just a single layer of CS may not form a firm shell to cover the protein core. Therefore, the loose CS shell could easily swell in the SGF leading to rapid protein diffusion due to the concentration difference. With two more layers of starchy polyelectrolyte deposition, the thicker shell significantly slowed protein diffusion in the SGF of the triplet-layer nanocapsule. The triplet-layer shell began to swell in the SIF and swelled to a greater extent in the SCF, causing protein release. For the quintet-layer nanocapsule, the increased number of layers prevented the shell swelling in the SGF and SIF and further slowed the protein release. However, in the SCF, the increasing *n* value resulted in the maximum degree of swelling of the starchy shell, speeding up the protein release. These results indicated that increasing the number of deposited layers of the pH-sensitive starchy polyelectrolytes is an appropriate way to control the release kinetics of nanocapsules, and so obtain the required release profile. In our study, the quintet-layer nanocapsule showed the best release properties for protein, and the specific reasons are further discussed below.

### 3.4. Structural Properties of Starch Polyelectrolytes and Quintet-Layer Nanocapsule in Simulated GIT

#### 3.4.1. Interaction between Starch Polyelectrolytes in Simulated GIT

**Figure 4:**
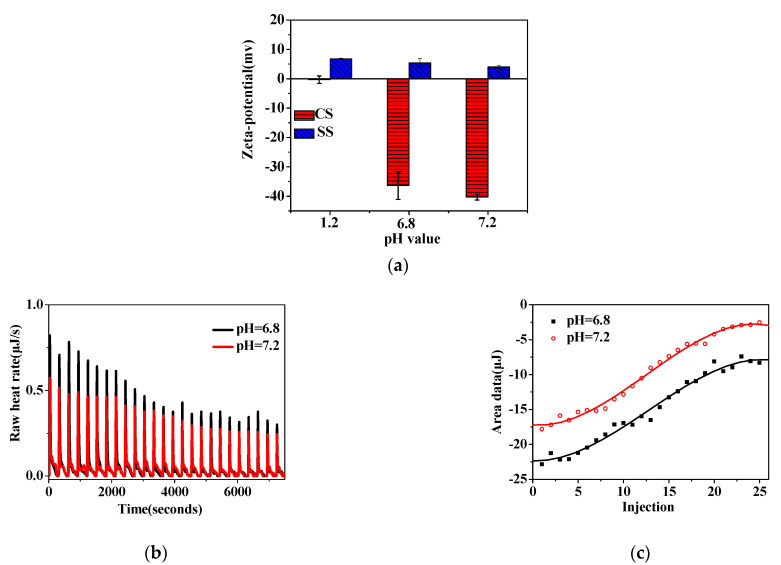
Zeta−potential (**a**) and integrated enthalpy (**b**,**c**) of the interactions between CS and SS at GIT pH. Abbreviations: CS, carboxymethyl anionic starch; and SS, spermine cationic starch.

After oral administration, the starch-based nanocapsule would pass through the stomach and small intestine and finally reach the colon. The surface properties of CS and SS presented pH-responsive behaviors (Figure 4a). The pH in the GIT ranged from 1.2 to 6.8 and reached about 7.2, leading to changes in the surface potential of CS and SS. With the increasing pH, the CS presented a more negative charge (Figure 4a). At pH 1.2, CS was neutrally charged because of the protonation of the carboxymethyl group. With the increasing pH, the CS was more negatively charged, caused by the rising deprotonation of the carboxyl group in the more alkaline pH. In contrast, the amount of positive charge of the SS showed a sustained decline (Figure 4a), which is because of the increasing protonation of amidogen on the spermine backbone with the rise in pH value.

At pH 1.2, the CS was slightly negatively charged, while SS and protein carried a positive charge (Figure 4a). In this case, there was weak interaction between CS (on the first, third, and fifth layers of the nanocapsule) and protein (core) or CS and SS (on the second and fourth layers of the nanocapsule). Furthermore, the outer CS layer remained in a gel-like state because of the protonation of its carboxymethyl group, preventing protein release. At pH 6.8 and 7.2, the interaction between CS and SS molecules was further elaborated using ITC. At pH 6.8 and 7.2, the negative enthalpy change (ΔH) and positive entropy change (ΔS) (Table 2 and Figure 4b,c) indicated that the interaction between CS and SS was a spontaneous exothermic reaction governed by electrostatic interaction [5]. The negative ΔG suggested that the interaction was exothermic and spontaneous. At pH 6.8, the binding affinity showed a higher value of 6.927 × 10^6^ M^−1^, indicating a stronger interaction between CS and SS in the SIF that improved the compactness of the shell, further slowing the release of protein in the SIF. In the SCF (pH 7.2), the binding affinity value (1.447 × 10^5^ M^−1^) was much lower than at pH 6.8, which showed a weaker interaction between the starch polyelectrolytes. As a result, the loose shell quickly swelled in the SCF to hasten protein release under the concentration difference. The required release profile could be obtained by controlling the deposited structure between the starch polyelectrolytes.

#### 3.4.2. Structural Properties of Quintet-Layer Nanocapsule in Simulated GIT

**Figure 5:**
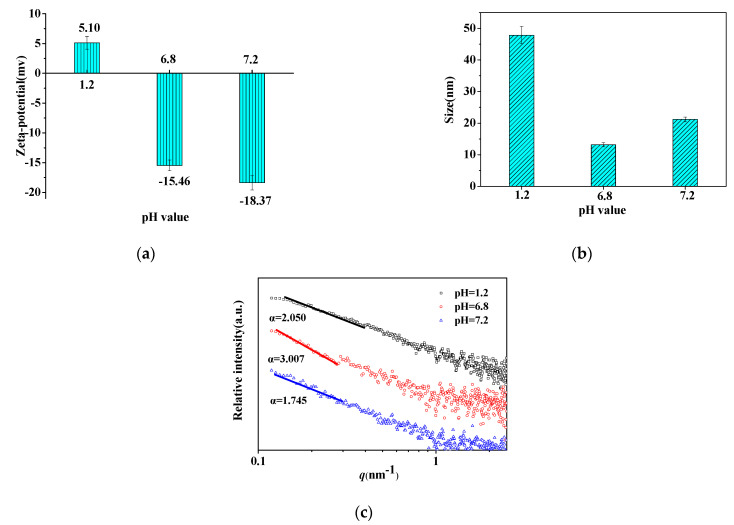
The surface potential (**a**), size distribution (**b**), and log I(q)−log q curve (**c**) of quintet−layer nanocapsule at GIT pH.

Structural changes of the quintet-layer nanocapsule should be closely related to the dynamic changes in the human GIT pH. The ζ-potential and size of the quintet-layer nanocapsule at different GIT pH values (SGF pH 1.2, SIF pH 6.8, and SCF pH 7.2) clearly showed that the delivery system was highly pH-responsive (Figure 5a). For the acid SGF pH, the nanocapsule was charged neutral, owing to deprotonation of the carboxyl group in the CS backbone on the outer shell. However, it was negatively charged in the SIF and SCF pH due to increasing deprotonation of the carboxyl group of CS and slight protonation of amidogen of SS on the spermine backbone. The negative charge increased due to the greater deprotonation of CS in the more alkaline SCF. This indicated that the CS quickly swelled in the SCF, helping the release of the protein cargo.

At the same time, the size of the quintet-layer nanocapsule tended to decrease in the SIF and increase in the SCF (Figure 5b). This is attributed to the joint result of the interaction between starch polyelectrolytes during the dynamic changes of GIT pH. Specifically, the CS was neutrally charged at pH 1.2 (SGF) because of protonation. In this case, the low repulsion between the nanocapsule made it easier to form a larger particle size. However, at the SIF pH (6.8), the surficial charge of CS (negatively charged) and SS (positive charged) would strengthen the electrostatic interaction between the layers of the quintet-layer nanocapsule to reduce its size. In this case, the compact shell effectively decreased the protein release rate. Furthermore, as pH rose to 7.2, carboxyl group deprotonation increased, while the degree of deprotonation of SS decreased. Therefore, the excessive negative charge increased the repulsion between CS and SS to increase nanocapsule size, thus hastening protein release.

The SAXS was used to characterize the aggregation structure of the quintet-layer nanocapsule for different simulated GIT pH values. According to the fractal characteristics, the aggregation structure of the quintet-layer nanocapsule at the GIT pH was determined, and a larger mass fractal dimension corresponds to a more compact structure [23,29]. The surface/mass fractal structure can be obtained from the slope of the log I(q)–log q SAXS graph. If the curve has a linear range, this shows that there is a fractal structure characteristic. The type of fractal structure can be judged by the value of the slope α. When 1 < α < 3, the ordered aggregation has a mass fractal structure. In addition, its fractal dimension is Dm = α, indicating the close degree of an orderly collective structure. The more significant Dm indicates a more compact structure of the ordered group. When 3 < α < 4, this indicates that the ordered aggregation has a surface fractal structure, and its fractal dimension is Ds = 6 − α, representing the degree of smoothness of the ordered aggregate surface [22]. The fractal dimension Dm of the quintet-layer nanocapsule was 2.050 in the SGF, 3.007 in the SIF, and 1.745 in the SCF (Figure 5c), which indicated that all the aggregation structures had a mass fractal structure. With pH rising from 1.2 to 6.8 to 7.2, the mass fractal dimension first increased and then decreased. In the SIF with pH 6.8, the mass fractal dimension reached a maximum of 3.007, indicating that it formed the most compact aggregation shell structure, which could inhibit protein release from the nanocapsule. However, the mass fractal dimension decreased to 1.745 in SCF. This indicated that the aggregation structure of the quintet-layer nanocapsule tended to be the loosest, making protein release in the colon easier.

## 4. Discussion

The release behaviors of protein cargoes from the nanocapsule in the GIT were mainly governed by shell structure. Therefore, scientifically constructing the deposited structure of starch polyelectrolytes on the basis of GIT dynamic changes is a sound approach to obtaining the required release profile of protein. The nanocapsule was designed with pH-responsive CS and SS as the outer layer and alternately deposited on the protein surface, to investigate how the number of deposited layers influenced the protein release properties and the release kinetics in the GIT, thus revealing the release mechanism of the quintet-layer nanocapsule.

As described above, the layer number of starchy polyelectrolytes (CS and SS) significantly influenced the structural properties in the dynamic GIT environment, regulating the in vitro release behaviors. Single polyelectrolyte deposition could not form a compact layer on the protein surface in the single-layer nanocapsule [30]. Although the CS had a gel state in the SGF, the stomach medium was quickly incorporated into the nanocapsule through the gap of CS. There was a rapid exchange of water and protein, resulting in a burst release along the concentration gradient (Figure 2). Subsequently, with the deposition of two more SS and CS layers, a relatively thicker triplet-layer shell was formed. The more layers, the narrower the diffusion channel [31], this significantly blocked the entry of the SGF medium and decreased protein release (Figure 2). With the increasing deprotonation of -COOH group (Figure 4a), there was strong electrostatic interaction between the CS and SS in the SIF (Table 2). However, the triplet-layer shell still could not resist erosion by the small intestine medium (pH and pancreatin) (Figure 3); there was quite a lot of protein release in the SIF. This slowed the protein release rate in the colon because of the smaller concentration gradient. The quintet-layer starchy shell showed a more suitable change in the dynamic GIT (Figure 3). The thicker and more compact quintet-layer shell increased resistance to enzyme in the upper GIT (Figure 5c), which could better protect the bioactive protein from the digestive medium [21]. However, the protein could be released quickly (Figure 2) with the increasing repulsion between the CS and SS (Table 2) and along a larger concentration gradient in the SCF.

**Figure 6:**
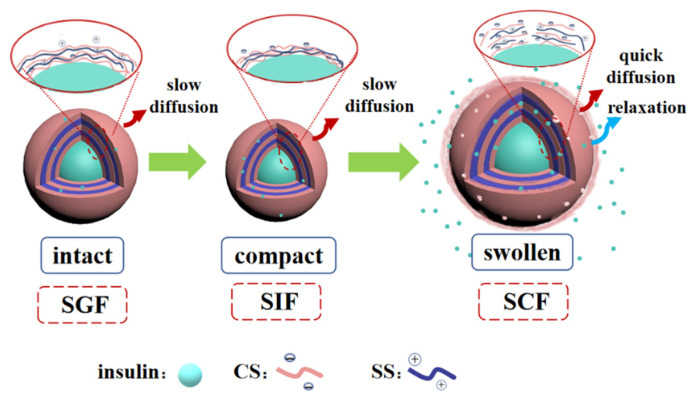
Relationship between controlled-release behavior and release kinetics of quintet-layer nanocapsule in the GIT. Abbreviations: CS, carboxymethyl anionic starch; SS, spermine cationic starch; SGF, simulated gastric fluid; SIF, simulated intestinal fluid; and SCF, simulated colonic fluid.

The schematic presentation (Figure 6) showed that the controlled-release mechanism of the quintet-layer nanocapsule in the GIT could be explained by the dynamic interaction of the starch polyelectrolytes in the dynamic GIT environment. The quintet-layer nanocapsule would sequentially pass through the stomach, and small intestine, and finally reach to colon during the oral administration. In the SGF, the quintet-layer nanocapsule surface tended to be gel-like because of great protonation of the carboxymethyl group on the CS interlaced on the surface and successfully prevented penetration of the gastric medium through the quintet-layer starchy network [19], resisting the exchange between protein and the gastric medium (Figure 5a). When transmitted into the small intestine, the CS presented a large number of negative charges due to deprotonation of -COOH groups in the SIF, strengthening the electrostatic interaction with SS (positively charged) (Table 2), constructing a compact aggregation structure (Figure 5c). Although protein release would occur due to the concentration difference, the compact shell significantly contributed to enzyme resistance in the SIF [19] (Figure 3). In this case, it effectively blocked the penetration of intestinal medium and pancreatin into the starchy network, decreasing protein release. Furthermore, with further transfer into the colon, the carboxyl group deprotonated to a greater extent (Figure 4a), while the degree of protonation of amidogen increased (Figure 4a). This weakened the interaction between CS and SS polyelectrolytes (Table 2). The quintet-layer nanocapsules showed a more negative charge (Figure 5a). The aggregation structure of the shell tended to be looser (Figure 5c), indicating the colonic medium more easily permeated through the swollen shell. Therefore, it enhanced the protein release under the high concentration difference (Figure 2 and Figure 3). To sum up, increasing the number of deposited layers of the pH-sensitive starchy polyelectrolytes is an appropriate way to adjust the release kinetics of nanocapsules, and, in turn, to appropriately control the release profile of protein in the GIT, thus achieving colon-targeted release.

## 5. Conclusions

In this work, the release behaviors of protein cargoes in the nanocapsules could be improved by increasing the deposited layers of pH-sensitive starchy polyelectrolytes. When increasing the layer number to quintet, the thicker and more compact starchy shell prevented the swelling of the nanocapsules in the upper GIT, simultaneously increasing repulsion between CS and SS in the colonic medium. As a result, this prevented the leakage of protein in the upper GIT and improved release properties in the SCF. Therefore, controlling the number of deposited layers of the pH-sensitive starchy polyelectrolytes on the nanocapsule is a helpful way to get appropriate release kinetics for colon-targeted delivery of protein cargoes in functional foods, thus acquiring the expected release profile.

## Figures and Tables

**Table 1:** Regression equation of drug-release kinetics of protein in the GIT.

Nanocapsule	GIT	Kinetic Models			
		Zero-Order Release Model		Peppas Release Model	
		K_0_ Value	R^2^	*n* Value	R^2^
Single-layer	SGF	34.267	0.965	0.606	0.966
	SIF	–	–	–	–
	SCF	–	–	–	–
Triplet-layer	SGF	4.007	0.975	0.403	0.994
	SIF	2.935	0.996	0.435	0.962
	SCF	3.405	0.993	0.732	0.971
Quintet-layer	SGF	3.232	0.999	0.369	0.994
	SIF	2.583	0.973	0.382	0.978
	SCF	3.692	0.981	0.787	0.994

Abbreviations: SGF, simulated gastric fluid; SIF, simulated intestinal fluid; and SCF, simulated colonic fluid.

**Table 2:** Thermodynamic parameters of the interactions between CS and SS assembled carrier materials at pH 6.8 and 7.2.

	pH 6.8	pH 7.2
Ka (M-1)	6.927 × 10^6^	1.447 × 10^5^
ΔH (kJ/mol)	−23.8	−15.2
ΔS (J/mol·K)	57.09	57.09
TΔS (kJ/mol)	14.16	14.13
ΔG (kJ/mol)	−9.64	−1.07

## Data Availability

The data presented in this study are available on request from the corresponding author.
